# Patient and caregiver perspectives of health provision practices for First Nations and Métis women with gestational diabetes mellitus accessing care in Winnipeg, Manitoba

**DOI:** 10.1186/1472-6963-14-440

**Published:** 2014-09-26

**Authors:** Hannah Tait Neufeld

**Affiliations:** Department of Geography, Western University, London, Ontario N6A 5C2 Canada

**Keywords:** Indigenous people, Canada, Diabetes, Health care disparities, Pregnancy

## Abstract

**Background:**

More North American Indigenous women are diagnosed with gestational diabetes mellitus (GDM) than the general population. Despite the number of health problems associated with GDM, few studies have been conducted that explore Indigenous women’s understandings of GDM in an effort to develop appropriate and effective health strategies.

**Methods:**

A qualitative investigation was conducted to describe the experiences of First Nations and Métis women with GDM. Unstructured interviews and focus groups initially took place with 25 advisors such as maternal care providers and community representatives. Semi-structured explanatory model interviews were subsequently carried out with 29 First Nations and Métis women in Winnipeg, Manitoba, Canada.

**Results:**

Divisions in health services, communication and cultural barriers exist, and limit prenatal care access as well as the consistent interpretation of diabetes education messages.

**Conclusions:**

Collectively the results suggest living with GDM can be overwhelming and underscore the need for health care providers to encourage self-efficacy towards effective management practices in the context of cultural safety.

## Background

The *Report of the Royal Commission on Aboriginal*^*a*^*Peoples* chronicled the lasting influence of past discriminatory policies on the lives of Indigenous peoples in Canada. One of its main recommendations was a call for justice and equity for all Canadians [1]. Eighteen years later, major discrepancies continue to exist in health status and health services access [2, 3]. Life expectancies for First Nations men and women are five to ten years lower than the Canadian averages and rates of chronic diseases such as heart disease and type 2 diabetes are significantly higher, with the prevalence of type 2 diabetes three to five times higher than the general population [4].

Indigenous women appear to be at significant disadvantage according to these health status measures with elevated rates of injury, suicide, obesity and diabetes relative to other Canadian women [5, 6]. Rates of gestational diabetes mellitus (GDM) are also higher [7–10]. First Nations women are at more than twice the risk of developing GDM, and have higher rates of pre-existing type 2 diabetes in pregnancy [4]. The overall health of First Nations women and their families continues to be impacted through assimilative action taken by the federal government to disconnect communities from their traditional lands and knowledge systems. These complex and continuing colonial policies have resulted in profound social and cultural disruption including the exploitation of lands, resources, and cultural practices [11]. Beginning in 1892, for example, greater than 150,000 children were removed from their families and communities and sent to schools operated by religious institutions and later the Canadian government [12]. The forced removal of these children to residential schools was intended to delimit the social and cultural identities of these children and acculturate them into Canadian society. The loss of language, ties to Elders, and traditional teachings isolated children from their cultural roots [13], which has had negative consequences for the health and well-being of many communities.

It is understandable under these circumstances that Indigenous families may continue to be distrustful of government-supported health care services in Canada. Studies describing the experiences and health needs of First Nations women and children have received less research focus proportional to their share of the population living in Canadian cities [14], yet young women tend to receive inadequate prenatal care [15]. In Winnipeg, higher rates of inadequate prenatal care were found in areas with the lowest family income, lowest levels of education achieved, highest percentage of single parent families and the highest percentage of the population reporting Indigenous background [15, 16]. A significantly higher proportion of these women receive insufficient care (15.7%) compared to 3.6% of non-Indigenous women [15]. Whether this is a result of lack of resources or perceived barriers to care has yet to be investigated in detail [16, 17].

There has been much discussion in the literature about culturally appropriate care or cultural safety for Indigenous populations associated with a general dissatisfaction with health care services on and off-reserve [18–22]. Browne and Varcoe [23] suggest that a “racialization” when it comes to health status indicators, or narrow conceptualizations of Indigenous ‘culture’ , as similarly described by Gray and Thomas [24], make it easy for health care providers to incorporate generalized assumptions into their practice, such as the negative perceptions of poor health and higher risk of diabetes and its visible complications. The model of prenatal care may not be perceived as ‘trustworthy’ because of the lack of adequate time to process or communicate women’s understandings of health messages [25–28].

Only a handful of previous studies have, however, examined the experiences of Indigenous women seeking prenatal care [21, 29–31]. First Nations women in British Columbia, for example, complained about “not feeling listened to” by their physicians resulting in a hesitancy to seek out mainstream health services ([21]; p.57). In a Pima community located in the southwestern United States, Smith-Morris [30] also found that a lack of trust in health professionals may result in patients making diagnostic or medication decisions for themselves. The anxiety associated with GDM diagnostic uncertainty may also force women to seek out external sources of information on the management of diabetes during pregnancy [28]. Health care services for First Nations women coping with GDM in an urban Canadian context has yet to be examined from the perspectives of both patient and caregiver.

Given the lack of formal investigation into this topic, a qualitative investigation was conducted with the aim of understanding First Nations and Métis women’s experiences with gestational diabetes in the urban context of Winnipeg, Manitoba. The primary objective in conducting this study was to describe how women experience gestational diabetes based on their own explanations of the illness or condition. This paper presents women’s perspectives on the prenatal care they received. The results are contextualized with the impressions of agency workers, Elders and health practitioners involved in First Nations and Métis women’s health in the city of Winnipeg to more completely describe the health care environment encountered.

## Methods

Agendas for Indigenous research constitute the potential for action that simultaneously involve processes of transformation, decolonization and healing of communities [32]. In pursuit of such dynamic research, it is critical to negotiate processes, approaches and methodologies. The most flexible and interpretive methodology to respond to an Indigenous research agenda is qualitative research. In-depth interviews are characterized by a more equitable balance of power with minimum control on the part of the researcher; the dialogue created is compatible with the politically motivated components of decolonization and self-determination [32, 33]. Participants are free to express themselves in their own terms. The elicitation of stories or narratives through the process of open-ended interviewing is concordant with Indigenous groups, whose stories, history and knowledge are based in oral tradition and experiential learning [32, 34–36]. Knowledge is highly rooted in practice, and traditional knowledge production requires the exchange of questions and answers [37], necessitating a quality of interaction that is more flexible and responsive to community needs [32].

### Research process

The range of in-depth interview formats utilized for this research study was therefore designed to provide a detailed description of Indigenous women’s experiences with GDM, as well as the health care services available in the city of Winnipeg, Manitoba. Data collection began with unstructured interviews or informal focus groups with a representative group of advisors^b^ that included maternal care providers, Elders and community leaders. The research design of this initial exploratory phase of the study was guided by grounded theory [38] as well as principles of community engagement [39] in approaching local practitioners and other knowledge holders. A sample of 25 individual advisors was initially contacted by letter asked to participate in either interviews or focus groups, utilizing the process of theoretical sampling [38]. After a series of informal meetings, four focus groups and six interviews took place with 10 sets of advisors. The focus groups were primarily conducted with health care professionals working in both clinical and community settings. One-on-one interviews occurred with two health care practitioners, two community advocates, one government employee and a female Elder. Five advisors self-identified as First Nation or Métis. Added description and pseudonyms are included for all the advisors in Table 1.

**Table 1:** **Advisor pseudonym, profession and Indigenous background (n = 25)**

Advisor pseudonym	Profession (Indigenous background)
Allison	Nurse manager
Amanda	Physician
Anne	Nurse
Barb	Diabetes educator
Brenda	Dietitian
Caroline	Nurse researcher
Cathy	Dietitian
Celeste	Midwife
Cheryl	Outreach worker (First Nation)
Denise	Indigenous women’s advocate (First Nation)
Emily	Dietitian
Heather	Dietitian
Holly	Midwife
Irma	Nurse
Isobel	Nurse
James	Physician
Jane	Nurse
Karen	Outreach worker (Métis)
Mary	Nurse
Megan	Nurse
Rebecca	Physician
Sophia	Social worker (Métis)
Susan	Dietitian
Suzanne	Prenatal program manager
Violet	Elder (First Nation)

Through these initial contacts with advisors, further support and participation from central and northend clinics, hospitals and local service agencies was established. Meeting informally with agency groups and attending community programs assisted in the recruitment of participants and created community awareness. Feed-back on the study approach and proposed objectives was also requested through informal discussions with individuals from representative organizations. The unstructured interviews and focus groups that took place with the 25 advisors explored issues and impacts of the health care system and other external support systems impacting prenatal care services, using questions that were formulated with collective input from the individual organizations approached. For example, health professionals were asked, “how do you think gestational diabetes affects Aboriginal women?” Community leaders and Elders were also invited to discuss and critique the availability of community support and resources for pregnant First Nations and Métis women in the city. These discussions provided context and support for the development of the semi-structured interviews that subsequently took place with participants who had experienced GDM.

The second series of interviews took place with a group of self-declared First Nations and Métis women who either currently had GDM or had been diagnosed within the past five years. These participants responded to posters displayed in various health care locations around the city. Informal presentations on the topic of GDM were made by the author to community and prenatal women’s groups to establish contact with potential participants. The author also regularly attended local antenatal and diabetes education clinics where women who fit the inclusion criteria were asked privately by nursing staff if they wished to participate in the study. A total of 30 participants were recruited using theoretical sampling [38]. One woman was excluded from analysis because it was later determined she had been diagnosed with type 2 diabetes when she was nine years old, which brought the sample size to 29. Twelve women self-identified as Saulteaux or Ojibway (Anishinaabe*)*. Nine women identified as Cree (Nêhinaw), two as Oji-Cree and four as Métis. Two others were unaware of their background since they had grown up in foster care. Additional background detail for participants appears in Table 2. Table 3 includes a complete listing of pseudonyms for the participants with added description.

**Table 2:** **Characteristics of First Nation and Métis participants (n = 29)**

Characteristic	Percentage of participants %
**Education level attained**	
Elementary school	7
Some high school	31
Completed high school	21
Some post-secondary	41
**Reported annual income**	
$5,000 – 15,000	31
$15,000 – 20,000	24
$20,000 – 30,000	24
$30,000 – 40,000	10
> $40,000	10
**Marital status**	
Married	17
Common-law	45
Single	38
**Occupation**	
Full-time caregiver	59
Unemployed	14
Private sector employee	17
Government employee	7
Student	3

**Table 3:** **Participant pseudonym, diabetes status, treatment, parity and residence (n = 29)**

Participant pseudonym	Diabetes status and treatment	Prior pregnancies	Residence
Alice	Type 2 after 4th pregnancy with GDM, 4 years ago; Insulin	5	Winnipeg
Amber	GDM with last pregnancy, 3 months ago; Insulin	1	Winnipeg
Anita	GDM with last pregnancy, 3 years ago; Insulin	4	Winnipeg
Carole^a^	Type 2 after 3rd pregnancy with GDM, 2 years ago; Insulin	3	Reserve
Cindy^a^	GDM; 2nd trimester; Insulin	2	Reserve
Claire	Type 2; GDM with last 2 pregnancies, 1 and 3 years ago; Insulin	2	Reserve
Darlene	GDM with last pregnancy, 3 years ago; Lifestyle	3	Winnipeg
Dawn^a^	GDM; Insulin	1	Winnipeg
Deb	GDM with previous 2 pregnancies, 1 and 2 years ago; Lifestyle	5	Winnipeg
Diane^a^	GDM with current and last pregnancy, 3 years ago; Insulin	4	Winnipeg
Doris^a^	GDM with current pregnancy; Insulin	0	Winnipeg
Elaine^a^	GDM with last pregnancy, 3 years ago; Lifestyle	1	Winnipeg
Eva	GDM with last pregnancy, 2 years ago; Lifestyle	3	Winnipeg
Faye	Type 2, GDM with last pregnancy, 2 years ago; Insulin	3	Winnipeg
Lena	GDM with last 2 pregnancies, 3 years and 5 months ago; Lifestyle	7	Winnipeg
Linda^a^	GDM currently and with last pregnancy 2 years ago; Insulin	4	Winnipeg
Lori^a^	GDM currently and with last pregnancy, 11 years ago; Lifestyle	1	Winnipeg
Lorna^a^	GDM with current pregnancy; Insulin	2	Winnipeg
Marion	Type 2 after 4th pregnancy with GDM, 1 year ago; Lifestyle	4	Winnipeg
Nancy	GDM with first pregnancy, 3 years ago; Lifestyle	2	Winnipeg
Pam	GDM with first pregnancy, 4 years ago; Insulin	2	Winnipeg
Sandy^a^	Type 2; GDM with last 2 pregnancies, 1 and 2 years ago; Insulin	3	Winnipeg
Sharon	GDM with first pregnancy 1 year ago; Lifestyle	1	Rural Manitoba
Stacey^a^	Type 2, GDM with last 2 pregnancies, 2 and 4 years ago; Insulin	4	Winnipeg
Terri^a^	GDM with all pregnancies; Insulin	3	Winnipeg
Tina^a^	GDM; Lifestyle	0	Reserve
Tracy^a^	GDM with previous pregnancy, 7 months ago; Lifestyle	10	Winnipeg
Tricia	GDM with last pregnancy, 6 months ago; Insulin	2	Winnipeg
Veronica^a^	GDM; Insulin	1	Winnipeg

^a^Pregnant at time of interview.

To guide these semi-structured interviews, Arthur Kleinman’s Explanatory Model Framework was used as a methodological orientation [40]. Because professional understandings of health tend to focus on the disease process, whereas personal experiences are often more complex in meaning [41], this orientation was chosen to complement the advisors’ perspectives and has been used to study illness experiences among other Indigenous groups in Canada [42, 43]. The theoretical assumption underlying this model is that people incorporate understandings as well as experiences into cultural frameworks and symbolic systems of meaning. Illness is culturally shaped in terms of how one perceives, experiences and lives with a condition or disease based on one’s own explanations. Explanatory models (EMs) are therefore subjectively and personally constructed, thereby reflecting the culturally situated meanings of an illness from the patient’s perspective [44]. These frameworks are viewed as cognitive beliefs created to recognize and respond to a specific illness experience, thereby potentially assisting with an individual’s ways of coping and making sense of one’s changing state of being [45].

These semi-structured interviews focused on the nature of women’s experiences with GDM and included a series of open-ended questions framed around five themes: onset, etiology, course of illness, treatment, and pathophysiology. Explanatory models generally contain explanations of five issues that inform the interview process and aid in the interpretation of the results [40]. The theme of prevention was added as a sixth theme to explore, given a previous lack of investigation into this area. Others have proposed that the framework is sufficiently flexible to incorporate the addition of wellness themes or concepts [46]. Sample questions can be found in Table 4. Quotes have been edited slightly to improve readability while not altering meaning [47]. Pseudonyms are used for the participants and advisors to preserve anonymity.

**Table 4:** **Sample explanatory model interview questions**

Illness category	Sample questions
Etiology	Do you think more women are getting gestational diabetes today than in the past? Why or why not?
Pathophysiology	If you had to explain to someone how gestational diabetes works, how would you describe what happens inside your body?
Onset	Can you tell me about how you found out you had gestational diabetes? When did it happen?
Course	Do you ever think that you will have diabetes again?
Treatment	How do you think diabetes during pregnancy should be treated?
Prevention	What do you think a woman can do so she will not get gestational diabetes?

### Analysis

The recruitment of advisors and participants took place from November 2006 until September 2007. During this period, interviews and focus groups were audio-taped and transcribed verbatim. During the entire research process, observational field notes were also kept. Transcripts were reviewed and corrected against the original audio files and an initial list of themes was incorporated into the project in NVivo7 [48] along with field notes. As recruitment continued to take place, transcripts from the advisors and then participants were read, searched and coded into categories and eventually themes, using a method of constant comparison across interviews and categories until data saturation was reached [49]. The process involved connecting emerging themes and modeling relationships by making memos to support the rationale and sequential process of the analysis until core categories were established and no new information was identified [50]. Member checking also took place at this stage of the research by consulting with both advisors and participants on the development of an analytical framework. These established themes and categories were subsequently further analyzed through matrix coding queries that assisted in categorizing relationships by linking participant characteristics or attributes with previously coded dialogue [51].

### Ethical considerations

Prior to data collection, ethics approval for the study was received from the University of Manitoba Health Research Ethics Board, the Health Sciences Centre Research Impact Committee, Health Canada Research Ethics Board, and the Winnipeg Regional Health Authority Research Review Committee. Written informed consent was obtained from all participants and advisors. Although not yet established at the time of data collection, the study approach was also consistent with the 2010 Tri-Council Policy Statement on research involving the First Nations, Inuit and Métis peoples of Canada [52]. The study began by engaging with community members and urban organizations, and involving local Elders and activists in the research process. Confidentiality agreements were strictly adhered to, along with OCAP research principles asserting the ownership of, control of, access to, and possession of the research results. The reporting of the qualitative methodologies employed in this study, also adheres to RATS guidelines for qualitative studies, in accordance with BioMed Central editorial policies.

## Results

Participants accessing health care services for gestational diabetes in Winnipeg ranged in age from 18 to 43 years of age. On average they had three children (range 0–10), and 16 were pregnant. Four women were living on-reserve, but travelling to Winnipeg for healthcare. All pregnant women are to be screened for GDM in Canada [4]. In Winnipeg this initial screening usually takes place with a general practitioner, obstetrician or midwife. Women who are diagnosed with GDM are encouraged to meet with an endocrinologist, diabetes nurse, and dietitian. First Nations women travelling into the city from nearby reserves commonly access these services at the Health Sciences Centre. Women from more remote First Nation communities generally attend prenatal and endocrine clinics based out of St. Boniface Hospital. Smaller community health clinics and centres in Winnipeg also provide education to women with GDM. Dietitians and nurses also provide individual counselling at these community sites as well as additional resources and health service referrals. Eighteen or 62% of participants in this study were prescribed insulin to manage their GDM. Nine women had GDM at the time of their interview, while 11 had experienced it previously. Nine had subsequently developed type 2 diabetes.

### Health service divisions

Two main categories arose from the interviews and focus groups with participants and advisors. The first category, ‘Health Service Divisions’ is made up of two themes: *‘*assumptions and impacts of blame’ and ‘burden of responsibility’*.* Both groups talked about barriers they felt limited access and quality of prenatal care along with diabetes education. Aspects of patient and caregiver communication that may compromise the delivery of prenatal care to First Nations and Métis women with GDM was also discussed, as illustrated in the following quote by Suzanne, a prenatal program manager with the provincial government:

I don’t know if it’s so much access to healthcare because I think in Winnipeg there would be access, but again it’s more trust of the system that there has been so much reporting done and things. And that they don’t want to be identified as people that have problems, right? I mean you’re always stigmatized as people that have all these challenges.

#### Assumptions and impacts of blame

For First Nations and Métis women with GDM seeking prenatal care in the city of Winnipeg, interactions with healthcare providers can also be negatively influenced by a lack of certainty when it comes to causation and risk. A diagnosis of GDM labels the pregnancy as being “at risk.” It may be assumed that women who develop GDM are not in control of their weight, level of activity or have poor eating habits. These assumptions may be reflected in the ways First Nations and Métis women are treated by their health care providers, as Rebecca, a physician from a Winnipeg clinic empathized:

There’s so much guilt and shame that women have put on them all the time over everything. And I think diabetes is another thing too because it’s not self-induced, but we tell them you have to watch your diet. You have to exercise. And then the women that come back and their sugars are still high. I think they’re worried that we blame them for not caring enough about their pregnancy or baby.

She recommended that, as health care providers, “we have to get over that hump and take the guilt away because that’s why they don’t come back to us. Maybe we lecture more than we listen.” Carolyn, a former nurse, described working at a diabetes clinic where she “did not care for the attitudes of the physicians that worked there.” She explained: “it was kind of a ‘blame the victim’ approach. [They] would say, ‘well, she didn’t stick to her diet, well why didn’t she?’ without any attention to all the various other problems that might be impacting on their ability to follow the diet.”

First Nations and Métis women feel these judgments in their interactions with health care providers, even though as Amber noted, “any woman can get it, it’s just more common in Aboriginal women.” Participants seeking care for GDM talked about feeling judged or looked down upon by their health care providers. Sharon recalled meetings with a dietitian: “The way that she was talking about the diabetes, kind of made me feel that it was my fault that I actually had it. Like that I wasn’t watching what I was doing to begin with, and this is how I developed it.” Darlene also had a negative experience. The dietitian she met with made her “feel like a child because she had her plastic apples and bananas and I was insulted by that. I’m 35 years old you know. Don’t insult me. I know it’s healthy and what’s not.”

The explicit blame and judgment of women with GDM in Darlene’s statements reveal were similarly described by Tina, who was living in a reserve community at the time. She talked about a good friend who had GDM with all of her pregnancies who was asked by a local nurse to talk to prenatal groups about her experiences. Tina provided this explanation as to why her friend decided not to participate:

In my community they don’t really judge people with it because we know that we could be getting it and we don’t know. But now a lot of people are starting to notice that gestational diabetes are not happening to older women. It’s happening to younger women now. Like say if my friend went around with the nurse and she could say her side of the story. But I guess she was too shy and too afraid that people would judge her because she had it right through all her babies and she had to take insulin. I think what people would judge her by is that she wasn’t eating right and everything, and blame her for it. But it’s not only her fault. I think it’s just passed down through our parents and they get it from their parents. It just goes from generation to generation, actually.

Comments made by Celeste, a midwife, further illustrate how health care providers’ frustrations can be misconstrued as blame or judgment. She described one of her First Nation patient’s lack of compliance as “poor attitude” by coming to her prenatal appointments “with juice cans and if you asked her if she changed her activity, or was eating in the way the diabetes nurse had talked to her: ‘well, no not really’.” Although her level of caring for her patient and baby is illustrated by Celeste’s obvious disappointment and worry, other advisors felt that health care providers may need to work on their level of understanding with First Nations patients. According to Cheryl, a First Nations woman working with a prenatal health agency, practitioners must be able to interact with tact, sincerity, compassion and respect. When asked how she felt current programming for pregnant First Nations women could be improved, she responded:

It’s all about attitude. A lot of times they’ll wear their doctor’s hat or their nurse’s hat or their professional hat and they use a lot of terminology that [women] can’t relate to. A lot of times I think if you come from a low place, you’re going to present as timid and shy. And if you’re already at that doctor’s appointment and you’re feeling that way, it’s kind of hard to hear what’s being said to you. Especially when you’ve got an attitude where somebody’s telling you [that] you have to do this and sometimes that information is presented in a really disrespectful way. And you feel that you’re being looked down at. So, I think attitude plays a huge part in terms of the kind of result you’re wanting to achieve with the individual.

#### Burden of responsibility

Contradictory education messages when combined with the “bureaucracy” of the healthcare system can lead to inconsistent service provision or divisions of responsibility in the clinical setting that often places the burden on patients. Women may not have their own established general practitioner or obstetrician in the city, especially if they are temporary residents or traveling into Winnipeg from reserve. With the exception of the four women living on-reserve at the time of the study, most participants were accessing prenatal services from smaller health centres or attended outpatient hospital clinics where there seemed to be less consistency or support. As one of the participants, Stacey lamented, “I don’t know what to think but I think that it’s because there’s so many different doctors seeing me, they all tell me something totally different*.*”

Another participant, Amber, talked about the lack of information when she went to the clinic and would see “maybe one pamphlet” on gestational diabetes. Because she was over 12 pounds when she was born she thought, “I was going to have a really overweight baby.” Her main source of information on potential outcomes and risks associated with GDM appeared to be almost exclusively based on personal experience, resulting in fear and anxiety to discuss her concerns with care providers or family. As she described her diagnosis with GDM: “it kind of was upsetting because I didn’t know what to expect, because you don’t hear a lot about it. We weren’t taught anything about it.” Doris complained about a lack of support in the city and suggested there should be “a lot more people going out and talking about [GDM] – the health reasons.” She went on to say, “you’re kind of shuffled around here. There’s some women who come down here [from reserve], and they don’t know anybody.” Although she had attended prenatal drop-in groups herself she found them to be more focused on “breastfeeding and your child’s temperament.” Gestational diabetes was “just not a topic that you really bring up.”

Several advisors mentioned barriers such as lack of time and resources prevented them from responding to First Nations and Métis women diagnosed with GDM. Health care providers at a Winnipeg hospital felt constrained over budgetary allowances and staffing. A nurse, Jane, explained: “the resources are there, but they’re very stretched. In terms of being able to, in an innovative way, create more time and other programs to support people with gestational diabetes. I don’t know if support groups or group classes would be beneficial.” She went on to suggest that perhaps, “we could improve on that by offering group classes or support groups. I think that that might be something that might be for another area or another organization to provide.”

Another advisor and prenatal program administrator, Suzanne, similarly commented on the peer group setting that “makes GDM easier to talk about”, but went on to say, “I think it’s just one of those things that it could be done with ‘here’s the brochure.*’*” The responsibility of dealing with the prenatal and postnatal challenges of GDM it appears can therefore be diverted to the pregnant patient or local clinics and agencies without sufficient support or explanation. This individualization of care can also extend from the clinical to the community setting. It is often up to women to attend community prenatal meetings for education and support while managing their own blood glucose and lifestyle regimes. Susan, a dietitian, commented that she and her colleagues at a hospital-based diabetes education centre often refer First Nations and Métis women with GDM to community agencies that specialize in prenatal care, although she was not sure how many patients attended: “some might phone and others they say they’ll do it. I don’t really know if they ever go or not.”

In the community as well as the clinical setting Carolyn, a prenatal health researcher and nurse, noticed that often women were receiving “different information about what to eat and the blood sugar readings they should be aiming for.” Even though there are benefits of having specialists, such as endocrinologists, manage conditions such as GDM, the entire health care team, “needs to know what the goals are so they’re giving the same message.” Unfortunately this may not be a reality as members of the healthcare team sometimes out of frustration with patient compliance, transfer GDM patients out of their care. Celeste, a midwife, responded immediately when asked what would happen if First Nations women were struggling to manage their GDM at her clinic. She exclaimed: “transfer them to OB!” Her colleague Barb, a diabetes educator, elaborated further: “obstetrics don’t see gestational diabetes as serious either and don’t refer them to an endocrinologist. Don’t continue sending them somewhere for education. I think that whole message of just the urgency and the seriousness of gestational diabetes and how serious it can be is not out there, with even a lot of the health care providers.”

Patients’ confusion as a result of inconsistent messages and attitudes can lead to instances of women who “fall through the cracks” or perhaps become apathetic about formalized care after a negative encounter. Under these circumstances women may be more susceptible to misinformation by isolating themselves, which can be particularly dangerous when it comes to managing diet and insulin. Faye knew that, “for anyone not familiar with adjusting the insulin amounts that they’ve been prescribed, it can wreak havoc.” Her endocrinologist, however, allowed her to adjust her own insulin. As she said, “I didn’t need his permission; I did it on my own. He wasn’t supervising my activity.” Lorna had a similar experience. After receiving mixed messages from a nurse and dietitian about her diet, she was relieved when her obstetrician told her, “you can eat anything you want when you are on insulin, just limit yourself*.”* She continued on to relay her doctor’s advice: “you’re the only one who could tell if your sugars are getting too high. You can up your insulin; just let me know when you up it.” Consequently, her insulin “started off at 4 [and increased to] 20 units during the day.” Her reaction was nonetheless positive. As she said, “it’s not bad. He kind of has me doctoring myself!”

### Patient and caregiver dynamics

The second main category ‘Patient and Caregiver Dynamics’ includes the two themes of ‘establishing trust’ and ‘leveraging compliance’. As Violet, an Anishinaabe Elder, expressed in the following quote, patient and caregiver dynamics require a thoughtful approach in establishing mutual understanding and trust between individuals:

I always ask myself, “who are we to judge?” I don’t know the pain of that person. Everybody has a story. They might have been traumatized in their childhood. And maybe they’ll never get to the place where their life is going to change. So they continue to live their choice. Whatever their choice is, I know that the Creator God understands it.

#### Establishing trust

The circumstances described in the previous section place First Nations and Métis women in a position where they feel they are required to navigate within the larger health care system without sufficient or consistent support. This added responsibility does not necessarily foster trusting or caring relationships between the health professional and patient. For instance, health care providers seemed to struggle with education messages in terms of the short-term and long-term health risks associated with GDM. Carolyn was conflicted in her opinion as a nurse: “how helpful it is to say that you’re going to be more at risk for diabetes in later life? That’s just an anxiety producer I think, unless you give women some of the tools and suggestions for what they need to prevent that from happening.”

For women who have already had GDM and delivered a healthy baby, they may not necessarily believe that complications could arise. Stacey, for example, was frustrated with the care she was receiving at the time of her interview. She was receiving conflicting messages from her care providers. She did not seem to know what to believe and stated: “they say [the diabetes] was there before I got pregnant and they say it’s going away. Then they say that I have to lose weight. And then, they say that any sore I get won’t heal. I don’t even want to listen to it anymore.” Diane was also confused and angry. She said, “gestational diabetes in general…I don’t know if it is dangerous ‘cause I’ve never been told if it is or it isn’t!” The dietitian she was seeing did not believe the written food records she kept were truthful because of her continued weight gain and told her, “how can I help you if you’re not honest with me?”

Several advisors were also aware of these communication issues. Health care professionals working in a diabetes education centre talked extensively about establishing trust with patients through support, mutual respect and reciprocity. A dietitian, Heather, explained: “they need to talk about things, which gives the level of trust [for them] to be more open and receiving to any recommendations that you might give because you’re looking at the whole person in totality and not just the disease.” From her perspective as a nurse, Jane described it this way:

I think that’s important in any therapeutic relationship. You have to understand where the person is coming from, what their perspective is. If you don’t take that minute or two to understand that information, then you’re just telling someone what they should think and should do. And your chances of success in terms of adherence, is probably lessened.

Participants also discussed the importance of taking the time to engage in a reciprocal exchange. Linda said, “my GP is just a fantastic doctor because he sits there and actually listens to his patients. He respects that they know as much about what’s going on with their body as he probably does, if not more.” Over half of the participants, however, had never heard of GDM when they were first diagnosed. Carole explained that she would have appreciated another perspective from someone who had experienced GDM and could “show you how it is, just to prove that the dietitian isn’t just there to tell you what to do.” Four other women also talked about the idea of a peer or support group for women with GDM to help them better understand its complexities and also to create more awareness in a culturally safe environment. As Sharon suggested, “because if you don’t know anyone else who’s had it, you don’t know how you’re feeling, if how you’re feeling is normal. If you’re talking to someone who hasn’t experienced it, they don’t know how you’re feeling.”

Sophia, a Métis community activist and social worker described similar circumstances based on her work with an inner-city social services agency. She explained that health professionals working in the “inner city” communities in Winnipeg do not have the same kind of credibility as another First Nations or Métis peer to identify with. As she stated, “you haven’t lived my life.” She went on to say, “I think overall you’re talking about a very disempowered community, so trying to come up with ways to empower them to think that they actually have control in their lives on a variety of factors is pretty challenging.” Sophia and an Elder, Violet both talked about the importance of trust and changing health professionals’ attitudes towards the strengths of lay teachers from the community to motivate women to move beyond their past and current circumstances. Violet talked about the benefits of healing circles, to speak to women not at a level of education, but instead, “go to the heart and the spirit of the individual….and perhaps they might develop the courage to speak about what is most pressing and stressful in their lives at the moment.”

#### Leveraging compliance

Interactions in a clinical setting with primarily health professionals who do not have a similar cultural background unfortunately do not always provide the opportunity for women to express themselves while coping with GDM. Several practitioners were aware of the potential for miscommunication under these circumstances. A nurse-manager, Allison talked about the delicate balance that needed to be maintained during her interactions with patients at the inner-city clinic where she worked. She said, “it’s a two-way street. You have to feel that there is something being received and given back, and that there’s a transaction happening. It’s more than just you sitting there and loading them with information.” Other health professionals working in a hospital setting spoke about the concept of “forced adherence”, or as Jane explained, “adherence facilitated through guilt, shame and ridicule.” In other words the fear or “scare tactics” that some health care providers use as leverage to gain compliance and reinforce hierarchy. A colleague of Jane’s, Heather, explained why their health care team doesn’t resort to such tactics: “they’re not going to come back if you do that. That’s sort of counter-productive.”

Power struggles between care providers were also described by participants as negatively impacting care and causing distress and confusion. As Stacey said, “they were too busy trying to scare me to listen to me*.*” Several participants conveyed their fear and anxiety about GDM that appeared to result from a lack of comprehensive information transferred from care providers. Consequently, some would tend to disengage from the clinical encounter, perhaps to emotionally manage the added stress. Anita already had so much to deal with in her life having lost a child to sudden infant death syndrome and raising two grandchildren in addition to her four children. She would often “forget” to ask questions when she saw her doctor for GDM following her son’s birth and said, “there are still questions that I’d like to ask about it too, like what does it do to your body? I see women with diabetes and they don’t have a leg, you know. Why’s that?” Lena also found the diabetes education she received complicated. She said, “I like reading, but I couldn’t get into it. So I can imagine what it’s like for other women that don’t know [how] to read, [are] afraid to ask questions, don’t want to be embarrassed.”

During their interactions with health professionals, some participants expressed that they felt socially responsible to act more “outgoing” during health care encounters in order to feel accepted and respected. Marion talked about this concept of needing to be “outgoing” in order to positively adjust to GDM recommendations and feel support and approval from care providers. Marion was not able to achieve this for herself, but recounted the experience of a close friend, “in her second pregnancy, she listened [to her doctor] and she was more outgoing and that. And she never had gestational diabetes in her second pregnancy although she had it in her first.” Other participants were more self-assured and assertive, as Lori urged: “we as patients should be asking, ‘well, why do we need to do that?’ We’ve got to ask questions.”

Both Linda and Faye were confident and knowledgeable as they talked about their experiences with GDM, as well as their interactions with health care professionals. Faye had challenged herself to successfully manage her GDM and the type 2 diabetes that developed postpartum. During her interviews she said she felt a need to impart a sense of independence and empower other First Nations women to confront gestational diabetes in a positive and powerful way. She wanted to know what was happening at every appointment and recommended to women: “even if he’s in a hurry he can answer a question. If you feel that there is something that’s very important for you to learn, you, as a patient, have a right.” Faye went on to suggest more flexibility when it comes to health promotion and lifestyle intervention:

I think that [a] very high proportion of people in general, when they’re diagnosed with a certain kind of illness, then they just accept that there isn’t anything that they can do about it. So I think definitely education, empowering people with knowledge of how they can manage their well-being. I think that’s a crucial factor in changing diabetes among not just Aboriginal communities, but all communities.

## Discussion

Participants and advisors both described the need for change in the delivery of health services for First Nations and Métis women with gestational diabetes. Rather than the more commonly cited barriers to care previously reported in the literature such as transportation, or logistical issues such as long wait times [53, 54], there appear to be other more complex factors at work. According to the group of advisors, divisions in health services as well as the dynamics of patient and caregiver interactions may place too great of a burden of responsibility on First Nations and Métis women to respond to and manage a pregnancy complicated by GDM without the appropriate amount of guidance and support from health professionals. Reasons for these circumstances may stem from inconsistent messages in diabetes education resulting from divisions in prenatal health services in Winnipeg. Structural inequalities also appear to exist that present ethical dilemmas and potentially contribute towards the rejection or displacement of care.

The distribution of prenatal care and support services to community agencies described by both advisors and participants accessing the healthcare system in Winnipeg can create an organizational structure that is not always accessible to First Nations or Métis women seeking care for GDM. Although community-based programs effectively fill in service gaps and reach women who would not normally seek out formal prenatal care, circumstances can also arise where women are managing their own care with inconsistent and conflicting messages between health care providers in community and clinical settings. Participants as well as other authors have described positive instances of self-care for women with GDM [25]. Administering insulin doses and managing a diabetic pregnancy however requires consistent and capable support as the nurse/researcher advisor, Carolyn, recommended.

Risk factors for type 2 and gestational diabetes have the potential to encompass negative stereotypes, as several participants alluded to in this study. Women may be viewed as lacking willpower, judgment or moral fortitude [55], which severely jeopardizes their self-image and makes them more vulnerable to personal attack and being discredited as “bad patients” or even “bad mothers” ([56]; p. 152). As described by several advisors, First Nations and Métis women with GDM can be morally judged and condemned for their assumed apathy towards lifestyle changes prescribed. These discriminatory attitudes of implied blame however, in themselves appear to contribute towards lack of compliance and poor attendance patterns. Similar results have been reported [28, 30]. According to Evans and O’Brien [28], the negative stigma associated with GDM is embarrassing for women and implies they are unhealthy.

Diabetes education messages were perceived by participants to be presented in a disrespectful way in this study. Several felt they were being blamed by health professionals for developing GDM. Lawson and Rajaram [57] similarly reported discriminatory practices against women with GDM, along with another study that described women as resentful and angry about the way diabetes education messages were delivered. Health care providers were viewed as disrespectful in light of their abruptness and seeming inattentiveness to patients’ concerns [27]. First Nations women accessing urban health and social services in British Columbia reported similar issues [21]. Women talked about feeling judged and described circumstances of racism. They described assumptions health professionals made about child neglect and alcohol abuse. Associating gestational diabetes with such negative stereotypes may permit health practitioners greater latitude to individualize its management, and in doing so limit their responsibilities for patient attendance and compliance. Bureaucratic divisions within the healthcare system appear to facilitate this with insufficient time or individual attention given during prenatal care visits, as was indicated by several participants in this study.

Historically Indigenous peoples have had many reasons not to trust dominant cultural groups, with past discriminatory policies continuing to negatively impact health [13, 53, 50, 58]. For women who have also been victims of abuse or had children apprehended, they may be even less likely to seek or trust formalized services, especially with the health and care of their families. Compromised communication patterns can parallel an underlying lack of trust. Pima women, for example, blamed a lack of cultural understanding for the misinformed perspectives of physicians and withdrawal of women through silence to avoid confrontation with an authority figure [30]. The same study noted how important a physician’s approach is for women to feel encouragement and support. Otherwise lower attendance rates may stem from a lack of understanding on the part of the patient, who may have an altered perception of health risks related to GDM. It takes time to develop trusting relationships and translate biomedical concepts into personally meaningful messages. Women who are told about their diagnosis with GDM may be offered no other explanation or support [57]. Attempts to assist women emotionally with a difficult pregnancy by shielding them from potential difficulties down the road not only brings up a myriad of ethical issues, it may backfire and further isolate them by contributing to the uncertainty and lack of understanding women in this study expressed when it came to their health and their baby’s health. Many are living in socially isolated environments to begin with. If they sense their health practitioner is not being honest with them, either by withholding information or not believing women’s personal experiences, a relationship of reciprocal trust cannot be formed. Benoit and colleagues [18] described the need for a safe, supportive environment for First Nations women in Vancouver. Other authors suggest, as the Elder Violet similarly recommended, the need for emotional process, time and understanding through healing circles to draw women out of their isolation [21, 27, 56].

Imbalances of authority or power were implied or described by participants and advisors that may negatively impact patient-provider interactions. A nurse Jane referred to the idea of “forced adherence” or scare tactics as a means of coercing compliance. Participants also talked about modifying their personality to find acceptance during health care encounters. Using blame to stigmatize, as described previously, can influence women’s behaviours by wielding power or reinforcing hierarchy. The “scare tactics” referred to by several participants and advisors in this study have been reported elsewhere as a method of coercing conformity. Participants in Adams’ qualitative study [59] among Latin American women with type 2 diabetes described similar incidents where scare tactics were used to promote compliance. Inciting fear in patients, however, is often the greatest motivator against accessing health services for Pima women with diabetic pregnancies [30]. Visual cues make it too easy for care providers to target and place pregnant women under surveillance [56]. Their position of vulnerability because of the pregnancy itself further isolates and victimizes women as persons “in need” [55]. This loss of autonomy and decision-making responsibility make First Nations and Métis women with GDM possible targets of exploitation as well [19, 23, 31, 60].

Under circumstances where the intolerance of unreasonable treatment becomes a negative personality trait [30], it is easier not to be “outgoing” as Marion described. It requires less interaction when one completely isolates themselves, or lives on the periphery of society, especially for First Nations women living in a large urban centre or travelling back and forth from their communities. The ability to exercise self-determination as described by Faye, should not at the same time compromise health, such as self-administering insulin doses. Being able to speak openly and participate in health care decisions through “shared knowledge and power” instead permits reciprocal knowledge exchange and can potentially contribute to personal empowerment ([19]; p.139). Having to gain credibility by transforming one’s behaviour or appearance as described in this study and others is not acceptable [19, 55], and to accept the responsibility for this transformation is to affirm judgments of inadequacy in the eyes of health professionals. It is a form of biculturalism, which in itself is a source of alienation or marginalization from mainstream culture and society [27].

## Conclusions

The experiences of First Nations and Métis women with the health care system in Winnipeg points to a need for change in the way prenatal care is delivered for those diagnosed with gestational diabetes. Clinical and community interactions need to be comprehensive, consistent and supportive. The shame and stigma associated with the label of GDM must also be dismantled through positive interactions, awareness and education. Practitioners need to present education messages in a positive, non-judgmental way with a focus on the concept of reciprocity, or knowledge exchange. Several participants expressed that they learned the most about GDM from experiencing it themselves. Providers must focus on this potential in terms of programming and effective self-management.

Even though health professionals follow ethical standards and feel interactions with patients are appropriate, many are seemingly unaware of how they may unintentionally demonstrate discriminatory attitudes. As a society, the majority of Canadians have a limited understanding of how colonial policies and practices have impacted First Nations communities in the way they perceive institutionalized health care [21, 61]. According to a Society of Obstetricians and Gynecologists of Canada (SOGC) policy statement, barriers facing First Nations individuals seeking health care include not only socioeconomic, but attitudinal, structural and communication barriers [62]. This statement has paved the way towards the establishment of evidence-based consensus guidelines for health professionals working with First Nations, Inuit and Métis [63]. SOGC recommends that in order to provide safe and adequate care, health professionals must navigate their interactions with skill, sensitivity, and awareness, recognizing the challenges of geography, historical trauma, cultural and linguistic differences, socioeconomic status, and well as persistent health inequalities.

Other professional associations, such as the Indigenous Physicians Association of Canada (IPAC) in collaboration with the Royal College of Physicians and Surgeons of Canada (RCPSC), are working toward changing these systemic barriers to care. Several years ago IPAC published comprehensive resources aimed at educating medical students and other health professionals on the importance of promoting culturally safe care for First Nations, Métis and Inuit patients [64]. The most recent Canadian Diabetes Association Clinical Practice Guidelines include a section on “*Type 2 Diabetes in Aboriginal Peoples*” that encourages culturally appropriate care through community partnership [4]. The Aboriginal Nursing Association of Canada (ANAC) has also been implementing strategies to increase in number and mentor First Nations, Métis and Inuit health professionals [65, 66]. As described previously, the Maori concept of cultural safety is unique and distinct from similar terminology used in health care settings such as cultural awareness, sensitivity or competence, aimed at acknowledging or respecting differences [22, 55, 61, 67, 68]. Cultural safety includes the practice of self-reflection, or a critical examination of power imbalances, which encourages a more patient-centred approach and the establishment of trust, respect as well as open communication [25, 26, 53].

It should be noted there are limitations in generalizing the results of this study to other First Nations and Métis women living in an urban setting or on-reserve. Results may not accurately describe the larger population of women living in Winnipeg. The majority of women recruited for the research were already accessing prenatal or diabetes care independently of this study. Nine women were interested enough in the topic to contact the author on their own. The experiences, therefore, of other First Nations and Métis women with GDM who are either not receiving regular prenatal or endocrine care are not included in these results. The group of advisors who freely participated in the study, also had an interest in the topic. There were other health care providers and agencies that were approached, but did not wish to take part formally. Given the small sample of advisors, their wide range of opinions are not necessarily representative of the collective group of providers and community advocates in Winnipeg.

This study, however, is the first to examine First Nations and Métis women’s experiences with GDM in an urban context, and in doing so identified several barriers to treatment and management. The results clearly illustrate the divisions of power and attitudinal barriers that without a doubt exist for First Nations and Métis women in the city of Winnipeg. Significant numbers of women are not seeking prenatal care which is a particular concern for those with complicated conditions such as GDM [15]. There is a need for a greater understanding as to why this is occurring because when faced with gestational diabetes, women living in a large urban centre are vulnerable and in need of trusted assistance and consistent support. Participants in this study expressed the desire for improved knowledge, increased awareness and the opportunity to share their experiences with GDM. A more reciprocal model of care is therefore proposed to empower women and their extended support systems through increased awareness and education. In short, a more positive experience is essential for First Nations and Métis women with GDM. Health care providers would similarly benefit from learning more about women’s life circumstances and challenges, as noted by several other authors in the context of type 2 diabetes [69, 70]. Elongated conversations between provider and patient allow the provider a voice in the patient’s translation of biomedical information into personally meaningful concepts [56]. This may require changes in policy at both the provincial and federal levels in Canada in the provision of prenatal care, as well as structural adjustments in the allocation of scarce resources.

Women play central roles for family and community. All women want to be accepted and viewed as good mothers, but according to the participants in the study, they do not necessarily receive these messages from those around them, especially when they feel scrutinized for the way they eat and live their lives. For the First Nations and Métis women who participated in this study, it is the health of their children they want and need to focus on and be proud of. They are trust their health care providers with their lives and the long-term health of their families. That trust needs to be respected and reciprocated in the context of cultural safety for mother and child.

## Endnotes

^a^In Canada, “Aboriginal” includes status and non-status First Nation, Inuit and Métis people [71].

^b^Use of the term “advisor” is used throughout this paper to replace the more common term of “key informant”, which according to numerous authors can be perceived negatively [32, 72].

## Author’s information

Hannah Tait Neufeld, PhD, PHEc, is currently a Banting Postdoctoral Research Fellow in the Department of Geography at Western University in London, Ontario, Canada.
